# Exosomal non-coding RNAs in autoimmune diseases: molecular mechanisms and potential applications

**DOI:** 10.3389/fimmu.2026.1753611

**Published:** 2026-02-19

**Authors:** Yunbing Wu, Xia Li, Yue Jin, Fei Mao, Lu Zhang

**Affiliations:** 1 Department of Laboratory Medicine, Northern Jiangsu People’s Hospital, Yangzhou, China; 2 Center of Molecular Diagnostic, Northern Jiangsu People’s Hospital, Yangzhou, China; 3 Medical Research Center, Northern Jiangsu People’s Hospital, Yangzhou, China; 4 Department of Laboratory Medicine, School of Medicine, Jiangsu University, Zhenjiang, China; 5 Department of Prenatal Diagnosis, Center of Medical Genetics, Yancheng Maternal and Child Health Care Hospital Affiliated to Jiangsu Medical College, Yancheng, China

**Keywords:** autoimmune diseases, exosomes, extracellular vesicles, intercellular communication, non-coding RNAs

## Abstract

Autoimmune diseases (ADs) are typically characterized by the immune system erroneously targeting self-organs and tissues, leading to chronic inflammation and organic damage. Current therapeutic strategies, which largely focus on suppressing late-stage inflammation, often fail to achieve satisfactory long-term outcomes. Exosomes, nanoscale extracellular vesicles secreted by diverse cell types, play a pivotal role in intercellular communication and immune regulation through the transfer of bioactive molecules, including non-coding RNAs (ncRNAs). Accumulating evidence indicates that exosomal ncRNAs, primarily microRNAs (miRNAs), long non-coding RNAs (lncRNAs) and circular RNAs (circRNAs), have emerged as critical regulators in the pathogenesis of ADs. They modulate key processes such as immune regulation, oxidative stress, autophagy and the cell cycle. In this review, we summarize recent advances in the regulatory mechanisms of exosomal ncRNAs in autoimmune diseases, with specific emphasis on Systemic Lupus Erythematosus (SLE), Rheumatoid Arthritis (RA), Type 1 Diabetes Mellitus (T1DM), Inflammatory Bowel Disease (IBD), and Sjögren’s Syndrome (SS). We also highlight their potential as diagnostic biomarkers and therapeutic targets. Finally, we discuss current challenges limiting clinical translation, such as delivery efficiency, stability, immunogenicity and large-scale validation, and propose future directions for the development of precision medicine strategies in autoimmune diseases.

## 1 Introduction

Autoimmune diseases (ADs) encompass a diverse group of disorders in which the immune system aberrantly targets self-tissues, disrupting immune homeostasis and driving chronic inflammation (1, 2). Affecting nearly 10% of the global population, ADs impose a substantial public health burden (3). Their onset and progression reflect multifactorial interactions involving aberrant antigen presentation, immune dysregulation, genetic predisposition and environmental triggers (4). Based on tissue specificity, ADs are broadly classified into organ-specific and systemic forms, with systemic autoimmune diseases (SADs) presenting heterogeneous, multi-organ manifestations and complex pathogenic mechanisms. Despite advances in understanding disease biology, early and accurate diagnosis remains challenging. Current approaches including symptom-based evaluation, autoantibody detection and invasive tissue biopsies, offer limited sensitivity for early-stage disease or dynamic monitoring. Furthermore, therapeutic strategies remain largely nonspecific (5). Thus, delineating the molecular mechanisms that govern ADs initiation and progression is essential for both mechanistic insight and therapeutic innovation.

Exosomes, nanoscale extracellular vesicles (EVs) released by diverse cell types, have emerged as key players in immune regulation and autoimmune pathogenesis (6). By transferring bioactive cargo, including proteins, lipids and ncRNAs such as miRNAs, lncRNAs and circRNAs, exosomes mediate intercellular communication and modulate both innate and adaptive immune responses (7, 8). Through this cargo transfer, exosomes influence antigen presentation, T-cell activation, cytokine secretion, and broader inflammatory pathways central to ADs development (9). For instance, exosomes from multiple sclerosis (MS) patients transfer let-7i to naive CD4^+^ T cells, suppressing insulin-like growth factor 1 receptor (IGF1R) and transforming growth factor β receptor 1 (TGFBR1) expression, thereby impairing regulatory T cell (Treg) differentiation (10). Beyond their pathogenic roles, exosomes also display unique advantages as therapeutic vehicles, exploiting membrane fusogenicity and surface adhesion molecules to achieve efficient, immune-compatible drug delivery.

However, it is crucial to acknowledge the complexities of EV nomenclature, as emphasized by the MISEV guidelines. Although exosomes are biologically defined by their endosomal origin, distinguishing them from other small EVs (sEVs) remains technically challenging due to overlapping physical properties and the limitations of standard isolation techniques like ultracentrifugation. Additionally, the lack of universal molecular markers often hampers the definitive characterization of specific subtypes. Therefore, consistent with the majority of the literature reviewed herein, we employ the term “exosome” to denote a broad range of EVs (11, 12).

Among exosomal cargoes, ncRNAs have attracted significant attention for their regulatory roles in immune homeostasis and disease. MiRNAs, short non-coding RNAs, post-transcriptionally suppress gene expression, orchestrating processes such as proliferation, differentiation, apoptosis and metabolism (13). LncRNAs (>200 nucleotides) regulate gene expression through transcriptional, post-transcriptional and epigenetic mechanisms (14), while circRNAs, defined by their covalently closed circular structure, exhibit exceptional stability and engage in transcriptional control, protein binding and signaling modulation (15). Increasing evidence implicates exosomal ncRNAs as critical regulators of immune dysregulation and inflammation in ADs. Aberrant ncRNA expression can exacerbate immune activation and tissue injury. Mechanistically, lncRNAs and circRNAs modulate immune networks by interacting with RNA-binding proteins, functioning as competitive endogenous RNAs (ceRNAs), and regulating signaling pathways such as NF-κB (16). For instance, in lupus nephritis (LN), IL-10 produced by CD8^+^CD103^+^ Treg cells suppresses lncRNA HAR1A expression, thereby attenuating NF-κB–dependent iNOS activation and ameliorating disease progression (17).

In this review, we synthesize current knowledge regarding the regulatory roles of exosomal ncRNAs in ADs, with a specific focus on SLE, RA, SS, T1DM and IBD. These conditions were selected as they represent the most prevalent phenotypes spanning the spectrum of both systemic and organ-specific autoimmunity, imposing significant clinical burdens. Distinct from previous broad overviews (18, 19), our analysis uniquely integrates emerging frontiers largely absent in prior summaries: the epigenetic regulation via N6-methyladenosine (m^6^A) modifications, the diagnostic utility of tRNA-derived small RNAs (tsRNAs), and the sex-specific pathogenicity of XIST-containing exosomes. Specifically, we highlight their contributions to immune dysregulation and inflammatory cascades, examine their potential as diagnostic biomarkers, and discuss emerging therapeutic applications. By integrating mechanistic insights with translational perspectives, we aim to underscore the promise of exosomal ncRNAs as both disease drivers and clinical tools, opening new avenues for precision diagnosis and targeted intervention in autoimmune disorders.

## 2 Exosomes: biogenesis, structure, and function

First identified in 1983 by R. M. Johnstone and colleagues in sheep reticulocyte cultures, exosomes (50–100 nm) are now recognized as essential mediators of intercellular communication and biomolecular transport (20). EVs are generally categorized into three groups based on size and biogenesis: exosomes (50–100 nm), microvesicles (100–1000 nm), and apoptotic bodies (1–5 μm) (21).

### 2.1 Molecular composition

Exosomes consist of a phospholipid bilayer enclosing a diverse repertoire of biomolecules, including proteins, lipids, nucleic acids and metabolites (22). Their lipid composition, rich in sphingolipids and cholesterol, ensures structural stability and functional versatility (23). Proteomic analysis identifies a conserved protein signature, including tetraspanins (CD9, CD63, CD81), heat shock proteins, major histocompatibility complex (MHC) molecules (MHC-I/II), transmembrane proteins (CD13, LAM1/2, PGRL) and various enzymes (23, 24). These proteins repertoire serves as a marker of the exosomes cellular origin and governs their interactions with recipient cells. These proteins not only reflect the cellular origin of exosomes but also mediate their uptake and biological effects on recipient cells.

Exosomes are particularly enriched in RNA species, particularly miRNAs. Other cargoes include small nuclear RNAs (snRNAs), ribosomal RNAs (rRNAs), transfer RNAs (tRNAs), lncRNAs, circRNAs and fragmented transcripts (25, 26). Importantly, exosomal mRNAs and miRNAs remain functional upon transfer, regulating gene expression in recipient cells and serving as a “molecular language” for intercellular communication (25, 27). Therefore, systematic profiling of exosomal cargo is pivotal for biomarker discovery and therapeutic development.

### 2.2 Biogenesis and secretion

Exosome formation is a highly orchestrated process that involves sequential endosomal maturation and membrane remodeling. Plasma membrane invagination generates early sorting endosomes (ESEs), which can also receive input from the trans-Golgi network (TGN), endoplasmic reticulum (ER) and mitochondria (28–30). ESEs mature into late sorting endosomes (LSEs) and ultimately multivesicular bodies (MVBs), formed by inward budding of the endosomal membrane. Most MVBs fuse with lysosomes for degradation, whereas a subset regulated by Rab27a/b and other exocytic machinery fuse with the plasma membrane to release intraluminal vesicles (ILVs) as exosomes (28, 31–33).

The molecular machinery underlying MVB and ILV formation primarily relies on the endosomal sorting complex required for transport (ESCRT) pathway (34). The canonical pathway involves four ESCRT complexes (0–III), the ATPase VPS4, and multiple accessory proteins, which act in a coordinated manner to cluster cargo and drive ILV budding. Non-canonical ESCRT-dependent mechanisms bypass upstream complexes by directly recruiting ESCRT-III and VPS4 to endosomal membranes (35, 36). In addition, ESCRT-independent mechanisms mediated by the melanosomal protein Pmel17, tetraspanin CD63, proteolipid protein and sphingomyelinase (SMase or SMPD2), contribute to ILV generation (33, 37, 38). Together, these complementary pathways constitute the molecular basis of exosome biogenesis.

### 2.3 Biological functions

Exosomes function as versatile messengers in both physiological and pathological contexts (39, 40). By transferring bioactive molecules reflective of their parental cells, exosomes participate in immune regulation, angiogenesis, tissue repair, proliferation, and inflammatory responses (41). Their functions are mediated through three principal mechanisms. Initially, ligand-receptor cognate recognition on the exosomal membrane facilitates intercellular communication via signal transduction. Subsequently, cargo deployment through membrane fusion with target cells enables macromolecular trafficking of genetic material and signature biomolecules. Finally, the release of proteins and miRNAs modulates target cell biology by engaging surface receptors, triggering intracellular signal transduction cascades that ultimately altering cellular function (42, 43).

**Figure 1:**
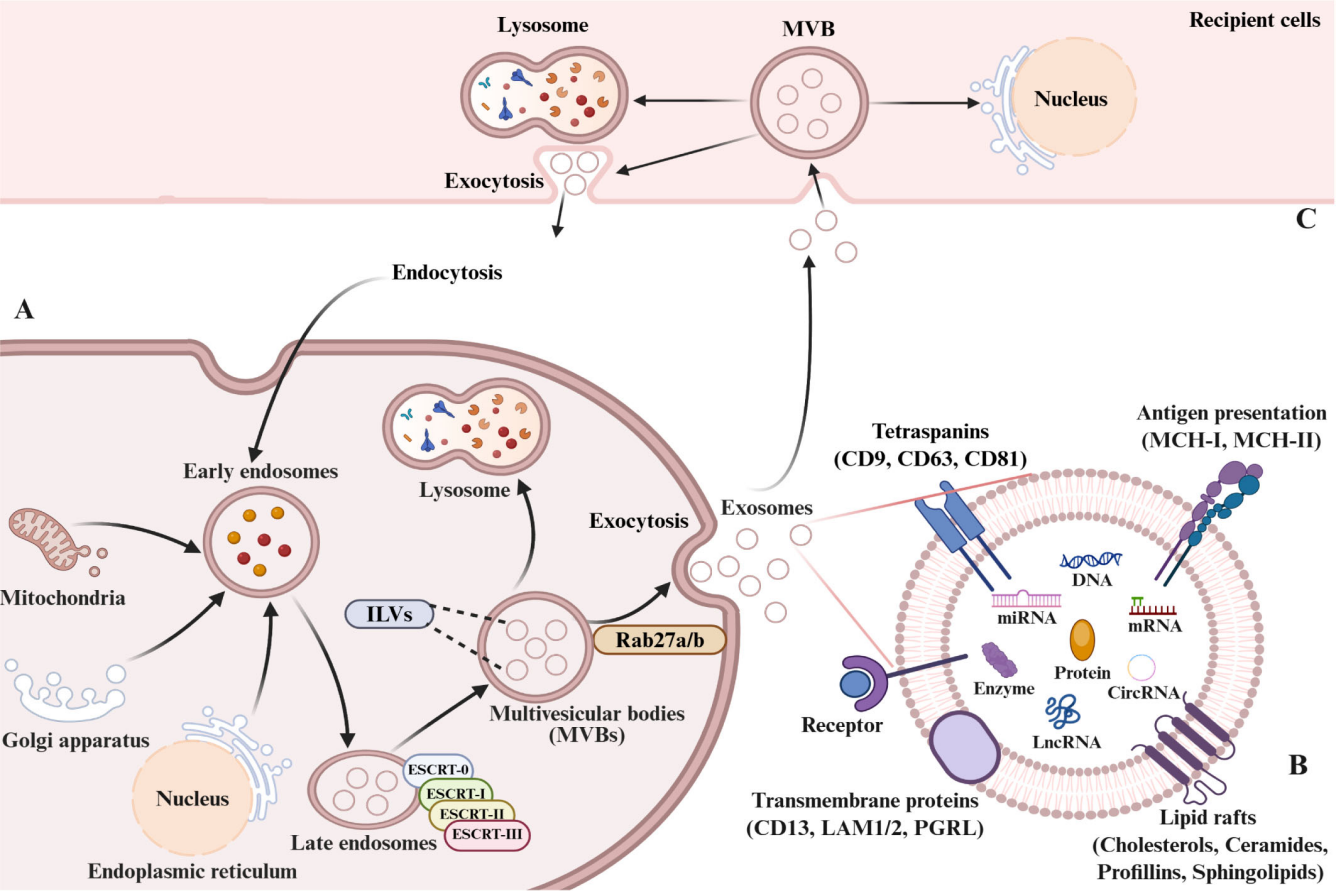
Exosome biogenesis, composition, and cellular uptake. **(A)** Biogenesis and secretion: Early endosomes form by inward plasma membrane budding. Vesicles from mitochondria, trans-Golgi network (TGN), or endoplasmic reticulum (ER) fuse with early endosomes. Invagination of late endosome limit membranes, couple with intraluminal cargo sorting, generates multivesicular bodies (MVBs). MVBs either mature into lysosomes or fuse with the plasma membrane, releasing intraluminal vesicles (ILVs) as exosomes. **(B)** Composition: Exosomal membranes are enriched in specific proteins (tetraspanins, integrins, transporters, immunomodulators). Luminal cargo contains proteins, nucleic acids (DNA, RNA), lipids, amino acids, and metabolites. **(C)** Uptake and fate: Recipient cells internalize exosomes via endocytosis or direct membrane fusion. Internalized exosomes mediate cargo delivery/signaling or undergo lysosomal degradation.

Collectively, exosomes represent highly specialized communication modules defined by distinctive biogenesis pathways and molecular signatures. Their stability, accessibility in body fluids and cargo specificity, underscore their potential as biomarkers for early diagnosis, prognostic monitoring, and therapeutic targeting in human disease (**Figure 1**).

## 3 Exosomal ncRNAs: mechanism of action in autoimmune diseases

High-throughput sequencing reveals that ~90% of the eukaryotic genome is transcribed into RNA, yet only ~2% encodes proteins (44). The majority of transcripts are ncRNAs, which emerge as critical regulators of gene expression and cellular processes. Mounting evidence indicates that ncRNAs, particularly those encapsulated within exosomes, contribute to the pathogenesis of multiple human diseases, including autoimmune disorders (23, 45). Exosomal ncRNAs mainly comprise miRNAs, lncRNAs, and circRNAs. Among them, miRNAs, short RNAs of ~20 nucleotides, suppress gene expression by promoting mRNA degradation or inhibiting translation (46). LncRNAs, defined as transcripts >200 nucleotides without coding potential, regulate gene expression at transcriptional, post-transcriptional and epigenetic levels (47–49). CircRNAs, generated through back-splicing to form covalently closed loops, display enhanced stability and often function as miRNA sponges, though they also participate in transcriptional and protein regulatory networks (50, 51). In this section, we summarize the mechanistic roles of exosomal ncRNAs in autoimmune disease pathogenesis, focusing on their regulation of immune responses, oxidative stress, autophagy, and cell cycle dynamics.

### 3.1 Immune response

Exosomes derived from immune and non-immune cells act as intercellular messengers, delivering ncRNAs that modulate immune regulation. Their immunomodulatory effects span immune cell crosstalk, T helper subset balance and macrophage polarization.

#### 3.1.1 Immune cell crosstalk

Exosomal ncRNAs shape immune cell activation and differentiation by mediating communication among immune subsets. For example, basophil-derived exosomal lncRNA ENST00000537616 promotes aberrant splenocyte proliferation and B cell activation in murine lupus (52). Activated T cell–derived exosomes transfer miR-146a into dendritic cells, targeting IRAK1 and TRAF6, thereby impairing antigen presentation (53). Similarly, M1 macrophage–secreted exosomal miR-155 exacerbates joint inflammation by reprogramming CD4^+^ T cell metabolism toward glycolysis (54).

Exosomal surface adhesion molecules further enhance cargo specificity. Psoriatic keratinocyte–derived exosomes transfer lncRNA AGAP2-AS1 to CD4^+^ T cells, activating the miR-424-5p/SGK1 axis to promote Th1/Th17 differentiation and psoriasis progression (55). Moreover, engineered mesenchymal stem cell (MSC) exosomes enriched in miR-146a attenuate pro-inflammatory cytokine release and restore T helper homeostasis in collagen-induced arthritis (CIA) models (56).

#### 3.1.2 Th17/Treg balance

Perturbations in the Th17/Treg axis are central to autoimmune pathology. Exosomal ncRNAs modulate this balance by targeting transcriptional networks. For instance, umbilical cord blood MSCs–derived exosomes (UC-BSCs-ex) deliver miR-19b to suppress KLF13 and restore Th17/Treg homeostasis (57). Exosomal miR-363-3p disrupts Treg function via the ARID3A/SPI1/TBX21 axis in immune thrombocytopenia (ITP) (58). Notably, MSCs-ex carrying ncRNAs exhibit significant immunomodulatory effects in autoimmune diseases by modulating the Th17/Treg balance. In SLE, adipose-derived stem cell derived exosomes (ADSC-ex) deliver miR-16-5p to downregulate LATS1, thereby restoring Treg function (59). Likewise, bone marrow MSCs (BMSCs) exosomal lncRNA TUG1 alleviates arthritis by targeting BLIMP1 and modulating Th17/Treg balance (60).

#### 3.1.3 Macrophages polarization

Exosomal ncRNAs also have the capability to influence the macrophages polarization. Macrophages constitute a heterogeneous population, broadly polarized into pro-inflammatory (M1) and anti-inflammatory (M2) subtypes contingent upon their activation state. Exosomes modulate macrophage polarization towards M1 or M2 phenotypes, representing a pivotal regulatory mechanism in inflammation that critically influences tissue homeostasis (61–63). In SLE, exosomal miR-122-5p promotes M1 polarization via NF-κB signaling by targeting FOXO3 (64), while serum exosomal let-7b-5p from Crohn’s disease patients drives M1 polarization through the TLR4 axis (65). Conversely, MSCs-derived exosomal ncRNAs favor M2 polarization, conferring protective effects. HucMSCs-ex enriched in miR-146a-5p downregulate NOTCH1, shifting macrophages toward an M2 phenotype and alleviating lung injury in lupus-associated alveolar hemorrhage (66). Similarly, BMSCs-ex carrying miR-16 and miR-21 promote M2 polarization via PDCD4 and PTEN targeting, thereby mitigating lupus pathology (67).

Collectively, exosomal ncRNAs orchestrate immune responses by modulating cell–cell communication, Th17/Treg dynamics, and macrophage polarization, underscoring their potential as immunomodulatory therapeutics.

### 3.2 Oxidative stress

Oxidative stress, characterized by excess reactive oxygen species (ROS), disrupts redox balance and promotes tissue damage in autoimmune diseases. Exosomal ncRNAs mitigate or exacerbate these processes. HucMSCs-ex carrying miR-140-3p suppress TNF-α and IL-1β production, reduce malondialdehyde, and enhance SOD activity in RA (68). Dysregulated exosomal miRNAs in RA, such as miRNA-103a-3p, miRNA-10a-5p, miRNA-204-3p, miRNA-330-3p and miRNA-19b, are linked to aberrant histone deacetylation and oxidative stress (69). In vitiligo, ROS-induced melanocyte-derived exosomes disrupt immune tolerance, whereas 3D-cultured human mesenchymal stem cells -derived exosomes (3D-hucMSCs-ex) enriched in miR-132-3p and miR-125b-5p restore Treg function and attenuate melanocyte injury (70, 71). Conversely, keratinocyte exosomal miR-31-3p exacerbates melanocyte destruction and CD8^+^ T cell activation under oxidative stress (72).

Furthermore, disruption of intestinal immune homeostasis leads to aberrant inflammatory responses and the accumulation of ROS. Consequently, targeted ROS clearance is a critical therapeutic approach. In IBD, oxidative stress impairs barrier integrity. Peroxiredoxin 3 (Prdx3) deficiency enhances colitis severity via exosomal miR-1260b–mediated intestinal barrier disruption and p38 mitogen-activated protein kinase (MAPK)/nuclear factor kappa B (NF-κB) activation (73). Together, these findings highlight oxidative stress as a key pathogenic mechanism modulated by exosomal ncRNAs across autoimmune contexts.

### 3.3 Autophagy

Autophagy constitutes an evolutionarily conserved intracellular degradation pathway that is essential for maintaining cellular homeostasis by degrading and recycling damaged organelles, misfolded proteins, and other cytoplasmic components. Under normal conditions, autophagy serves as an adaptive mechanism that promotes cell survival by mitigating cellular stress and maintaining energy homeostasis. However, excessive or dysregulated autophagy can induce cell death, characterized by distinct morphological hallmarks that may contribute to disease pathogenesis (74). Exosomal ncRNAs modulate this process, linking autophagy to autoimmune disease pathogenesis. Exosomal miR-20b-3p alleviates diabetic peripheral neuropathy by targeting STAT3 and restoring Schwann cell autophagy (75). Similarly, adipose MSC exosomal miR-486 inhibits smad1, suppresses mTOR activation, and enhances podocyte autophagy in diabetic nephropathy (DN) (76). In Crohn’s disease, intestinal epithelial cells derived exosomes (IECs-ex) deliver miRNAs that restore ATG5/ATG16L1 expression, counteracting bacterial inhibition of autophagy (77). Circulating exosomal miR-376a-3p and miR-20a-5p also regulate systemic autophagy pathways in Crohn’s patients (78). Furthermore, In SLE, altered plasma exosomal miR-20b-5p and miR-181a-2-3p promote apoptosis and autophagy in renal cells, aggravating lupus nephritis (79), while exosomal miR-20a modulates the mTOR pathway to attenuate renal injury (80). These studies position exosomal ncRNAs as dual modulators of autophagy, either protective or pathogenic depending on disease context.

### 3.4 Cell cycle dysregulation

Exosomal ncRNAs critically modulate cell cycle, specifically proliferation and division in autoimmune disorders, mechanistically driving pathological progression via dysregulation of cell cycle. In RA, plasma exosomal circ_0003914 promotes fibroblast-like synoviocyte (FLSs) proliferation and cytokine secretion via NF-κB/p65 activation (81), while TNF-α–stimulated FLSs.

-ex enhance angiogenesis in endothelial cells through the miR-200a-3p/KLF6/VEGFA axis (82). By contrast, MSCs-ex deliver miR-451a or circFBXW7 to suppress FLSs proliferation and inflammation, alleviating joint damage in arthritis models. MiR-451a-loaded hucMSCs-ex attenuated RA progression by targeting activating transcription factor 2 (ATF2), thereby suppressing synovial fibroblast proliferation, migration, and invasion, while ameliorating joint inflammation and radiographic abnormalities in CIA rats (83). Exosomal circFBXW7 suppressed proliferation, migration and inflammatory response of RA-FLSs and release the activation of HDAC4, thereby reducing joint damage of RA rats (84).

**Figure 2:**
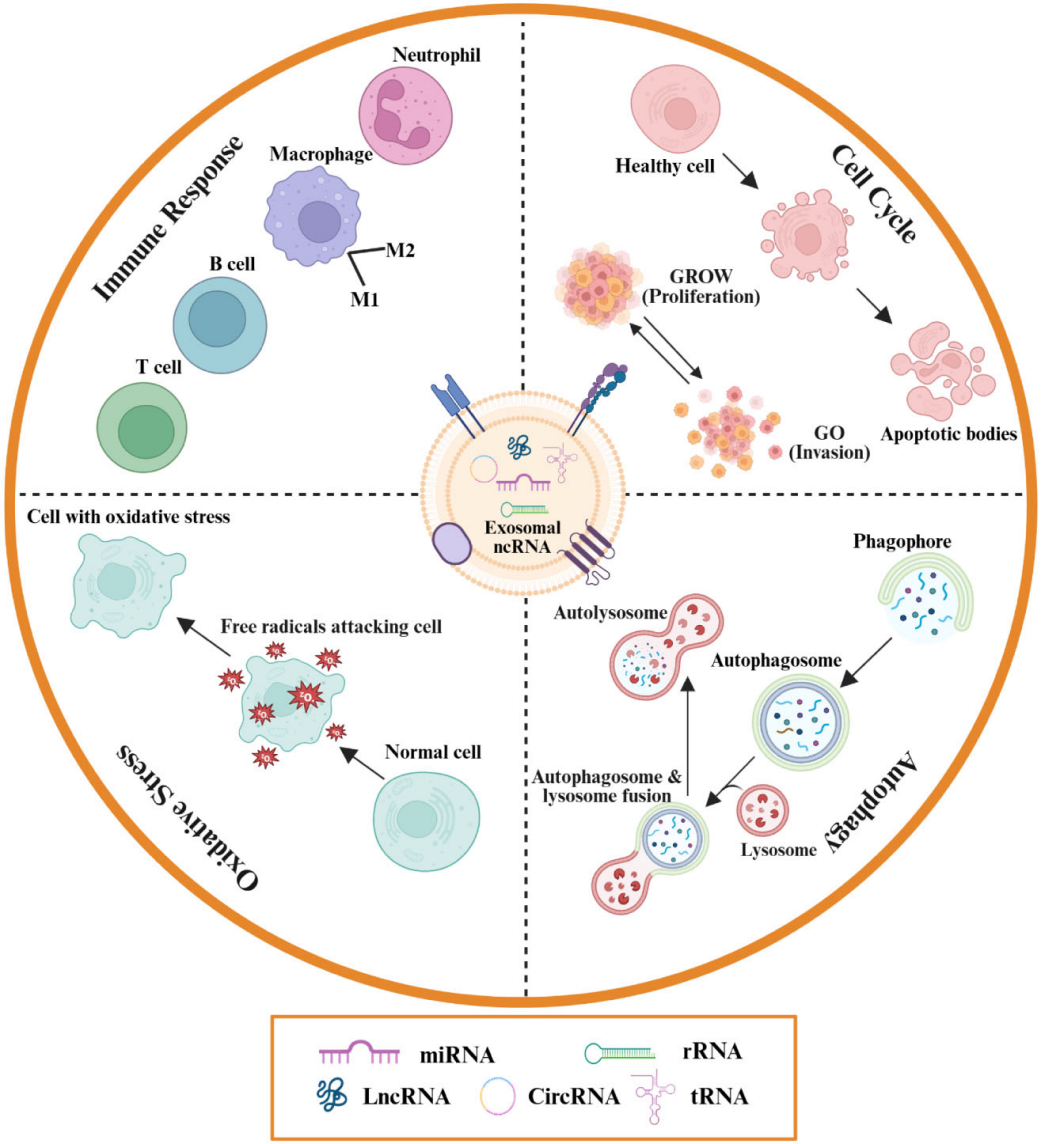
Exosomal ncRNAs mediate autoimmune disease pathogenesis through four principal pathways. They modulate immune responses by facilitating immune cell activation, antigen presentation, and cytokine secretion, thereby promoting or suppressing autoimmunity. Exosomal ncRNAs also regulate oxidative stress via cargo enzymes and molecules that influence the reactive oxygen species (ROS)-antioxidant balance, contributing to tissue protection or damage. Furthermore, they interact with autophagy pathways by delivering regulatory proteins and RNAs that control the degradation of damaged cellular components, impacting homeostasis. Finally, exosomal ncRNAs affect cell cycle control by delivering molecules that alter proliferation, apoptosis, and invasion, leading to aberrant immune cell behaviors characteristic of autoimmunity.

In addition, it was demonstrated that overexpression of exosomal miR-142-3p/−5p and miR-155 derived from T cells was sufficient to induce β-cell death, suggesting their contribution to the progression of T1DM (85). Whereas Treg-derived exosomes carrying miR-195a-3p support epithelial proliferation and reduce inflammation in colitis models (86). Thus, exosomal ncRNAs exert dual, context-dependent effects on cell cycle regulation, amplifying pathology in some settings while restraining it in others (**Table 1****) (****Figure 2**).

**Table 1:** Involvement of exosomal ncRNAs in the pathogenesis of Ads.

Function	Disease	Source of exosomes	Composition	Biological functions	Ref
Immune response	SLE	Basophilic	LncRNA ENST00000537616	Promote B cell activation	(52)
	RA	M1 macrophage	miR-155	Promote CD4^+^ T cell activation/differentiation	(54)
	Psoriasis	HaCaT	LncRNA AGAP2-AS1	Promote Th1 and Th17 cell differentiation	(55)
	RA	MSC	miR-146a	Attenuate pro-inflammatory cytokine production and restored Th subset homeostasis	(56)
	SLE	HucMSC	miR-19b	Regulate the Th17/Treg balance	(57)
	ITP	Plasma	miR-363-3p	Suppress Treg function	(58)
	SLE	ADSC	miR-16-5p	Restore the Th17/Treg balance	(59)
	RA	BMSC	LncRNA TUG1	Regulate the Th17/Treg balance and alleviates RA damage	(60)
	SLE	Plasma	miR-122-5p	Promote M1 polarization	(64)
	IBD	Plasma	let-7b-5p	Promote M1 polarization	(65)
	SLE-associated DAH	HucMSC	miR-146a-5p	Inhibit M1 and promote M2 macrophage polarization	(66)
	SLE	BMMSC	miR-16, miR-21	Promote M2 macrophage polarization	(67)
Oxidative Stress	RA	HucMSC	miR-140-3p	Promote SOD activity	(68)
	RA	Plasma	miR-103a-3p, miR-10a-5, miR-204-3, miR-330-3, miR-19b	Heighten histone deacetylation and oxidative stress	(69)
	Vitiligo	HucMSC	miR-132-3p, miR-125b-5p	Suppress oxidative stress-induced melanocyte damage	(71)
	Vitiligo	Keratinocytes	miR-31-3p	Destruct melanocytes and activate CD8 T cells	(72)
	IBD	IEC	miR-1260b	Promote intestinal barrier disruption and inflammation	(73)
Autophagy	Type 1 diabetes	Plasma	miR-20b-3p	Ameliorate autophagic impairment in Schwann cells, improve peripheral neuropathy	(75)
	Type 1 diabetes	ADSC	miR-486	Promote autophagic flux and inhibit apoptosis	(76)
	Crohn	IEC	miR-30c, miR-130a	Inhibit autophagy-mediated intracellular AIEC clearance	(77)
	Crohn	Peripheral blood	miR-376a-3p, miR-20a-5p	Regulate systemic autophagy and inflammatory pathways,	(78)
	LN	Plasma	miR-20b-5p miR-181a-2-3p	Promote HK2 cells apoptosis and autophagy	(79)
	LN	ADSC	miR-20a	Improve the prognosis of SLE	(80)
Cell cycle dysregulation	RA	FLS	hsa_circ_0003914	Promote proliferation, migration, invasion	(81)
	RA	FLS	miR-200a-3p	Promote HUVECs migration, invasion, and angiogenesis	(82)
	RA	Huc-MSC	miR-451a	Suppress proliferation, migration, invasion	(83)
	RA	MSC	circFBXW7	Suppress RA-FLSs proliferation, migration and inflammatory response	(84)
	T1DM	T cell	miR-142-3p/-5p, miR-155	Induce β-cell death	(85)
	IBD	Treg	miR-195a-3p	Promote cell proliferation and inhibit apoptosis	(86)

In summary, exosomal ncRNAs do not function in isolation but orchestrate a complex network to regulate autoimmune pathogenesis. Functionally, they orchestrate immune homeostasis through mediating immune cell crosstalk, balancing Th17/Treg subsets, and directing macrophage polarization (M1/M2 shift). However, their impact exhibits a clear duality primarily dictated by their cellular origin, target molecules, and the disease microenvironment (87, 88). Pathogenic exosomal ncRNAs, predominantly derived from activated immune cells or damaged parenchymal cells, drive autoimmune progression by disrupting immune tolerance, promoting pro-inflammatory phenotypes and inducing tissue damage. In contrast, protective exosomal ncRNAs, mostly derived from MSCs, alleviate autoimmune pathology by restoring immune homeostasis, suppressing oxidative stress and regulating autophagy.

Mechanistically, the four categories discussed (immune response, oxidative stress, autophagy and cell cycle regulation) are deeply interconnected. Exosomal ncRNAs regulate redox balance by altering ROS production and scavenging, modulate autophagic flux to maintain cellular homeostasis or induce cell death, and disrupt cell proliferation/differentiation cycles in disease-relevant cells. Crucially, these pathways often form a positive feedback loop: exosomal ncRNA-induced pro-inflammatory activation boosts ROS production, while excessive ROS further amplifies immune cell infiltration and cytokine secretion, exacerbating autoimmune pathology (72, 73). Furthermore, these mechanisms often converge on shared signaling hubs. Notably, the NF-κB signaling pathway serves as a common downstream mediator across multiple mechanisms, integrating signals from exosomal ncRNA-regulated immune response, oxidative stress and autophagy. For instance, exosomal miR-122-5p promotes M1 macrophage polarization via NF-κB activation in SLE, while circ_0003914 activates NF-κB/p65 to drive FLSs proliferation in RA (64, 81). This pathway convergence highlights potential therapeutic targets for simultaneous intervention in multiple pathogenic processes.

## 4 Roles of exosomal ncRNAs in the pathogenesis of specific autoimmune diseases

### 4.1 Rheumatoid arthritis

RA is a chronic autoimmune disease driven by persistent synovitis, bone erosion, and cartilage destruction, leading to irreversible joint deformities and disability (89). Although the pathogenesis of RA is not fully understood, current evidence indicates that genetic variants, dysregulated protein translation and post-translational modifications, and various environmental factors contribute to the onset and progression of the disease (90). Exosomal ncRNAs also emerge as key mediators in rheumatoid arthritis pathogenesis, actively contributing to inflammatory cascades under defined pathological states.

FLSs serve as pivotal orchestrators in rheumatoid arthritis pathogenesis. Their dysregulated activation and proliferation drive the secretion of inflammatory mediators (e.g., CXCL8, IL-6, IL-15), concomitant upregulation of adhesion molecules, and excessive production of extracellular matrix components such as fibronectin. These pathological alterations collectively potentiate leukocyte activation and recruitment into synovial microenvironments (91, 92). Exosomal circRNA hsa_circ_0003914 from RA plasma promotes aberrant FLSs proliferation and cytokine release through NF-κB/p65 signaling (81), while FLSs-derived exosomal miR-424, induced by synovial hypoxia, inhibits Treg differentiation and enhances Th17 expansion, tipping the Th17/Treg balance toward inflammation (93). Moreover, exosomal cargo also fuels synovial angiogenesis, with vascular endothelial growth factor (VEGF) and miR-200a-3p supporting hyperplasia and immune cell infiltration (82).

Beyond synovial inflammation, exosomes impair bone and cartilage homeostasis. The inhibition of osteoclast differentiation, proliferation, and mineralization constitutes a demonstrated mechanism that exacerbates disease progression. RA-FLSs exosomal miR-486-5p suppresses osteoblast proliferation and mineralization by targeting BMP/Smad–Tob1 signaling (94). Similarly, FLSs-derived exosomal lncRNA TRAFD1-4:1 sponges miR-27a-3p to upregulate CXCL1, driving extracellular matrix degradation and chondrocyte dysfunction.

Emerging evidence demonstrates that exosomal ncRNAs also contribute to RA pathogenesis by modulating immune cell polarization. Circ-CBLB–enriched exosomes activate TLR3/TRAF3 signaling to promote M1 polarization, while m^6^A modifications enhance circ-CBLB expression, underscoring the epigenetic regulation of inflammatory cascades (95).

### 4.2 Systemic lupus erythematosus

SLE is characterized by autoreactive lymphocyte activation, autoantibody production, and immune complex deposition, driving multi-organ injury (96). Among the complications of SLE, LN remains the leading cause of morbidity and mortality (97). During the incipient phase of SLE, exosomes regulate pathological immune activation through coordinated delivery of self-antigens and immunostimulatory molecules, thereby potentiating maladaptive recognition and targeted destruction of host tissues by autoreactive immune components. Exosomal miR-451a, enriched in CD4^+^ T cells and B cells, augments autoreactive responses (98).

A pivotal feature of SLE pathogenesis involves dysregulated B-cell activation, which drives excessive autoantibody production and inflammatory responses, ultimately culminating in tissue damage. Activated basophil-derived exosomes deliver lncRNA ENST00000537616, which sponges miR-330-5p to activate KRAS signaling, thereby potentiating B cell activation; silencing this lncRNA attenuates hyperactivation and improves renal pathology in murine lupus (52). Moreover, Senescence of MSCs plays a critical pathogenic mechanism contributing to the progression of SLE. Exosomal miR-146a mitigates MSCs senescence by targeting TRAF6/NF-κB, restraining disease progression (99).

Exosomal ncRNAs are also key drivers of renal pathology. Studies demonstrate that miR-20b-5p in plasma exosomes from patients with SLE mediates the promotion of apoptosis and autophagy in renal epithelial cells, thereby contributing to renal injury and the progression of LN (79). Plasma-derived exosomes from patients with SLE were found to accelerate disease progression and renal macrophage polarization in MRL/lpr mice. MiR-122-5p, upregulated in patient-derived exosomes, correlates with disease activity and dsDNA titers, promoting M1 macrophage polarization via FOXO3/NF-κB signaling (64). Furthermore, urinary exosomal miR-21 and let-7a activate NF-κB signaling in mesangial cells, amplifying inflammatory and fibrotic responses in LN (100).

### 4.3 Type 1 diabetes mellitus

T1DM results from T cell–mediated β-cell destruction, often presenting with diabetic ketoacidosis in children and adolescents. Exosomal ncRNAs modulate both beta-cell vulnerability and immune activation. For example, T cell–derived exosomes carrying miR-142-3p, miR-142-5p, and miR-155 induce β-cell apoptosis and upregulate chemokines (Ccl2, Ccl7, Cxcl10), facilitating immune recruitment (85). In addition, the pathogenesis of T1DM is characterized by insulin deficiency or dysfunction, leading to impaired glucose uptake and subsequent hyperglycemia. Adipocyte-derived exosomal miR-138-5p suppresses insulin secretion via SOX4/Wnt/β-catenin signaling (101).

T cells as pathogenic effector cells in TIDM are also well established. A fundamental pathological process in T1DM is characterized by the infiltration of activated T lymphocytes into the pancreas, inducing insulitis and the consequent loss of β-cells. A recent study demonstrates that MSCs-derived exosomal miR-25 reduces pancreatic infiltration of activated T cells by downregulating CXCR3, thereby alleviating insulitis (102).

### 4.4 Inflammation bowel diseases

IBD, comprising Crohn’s disease and ulcerative colitis, arises from the interplay of genetic susceptibility, microbial dysbiosis, and immune dysregulation (103). Exosomes serve as critical vehicles for ncRNA delivery, contributing significantly to the multifactorial pathogenesis of IBD. Exosomal ncRNAs influence disease through macrophage activation, epithelial barrier integrity, and host–microbiota interactions.

Macrophages are central to intestinal immune homeostasis, and their dysregulation fuels chronic inflammation. NLRP3 inflammasome activation-induced macrophage pyroptosis is implicated in the aberrant immune response underlying IBD pathogenesis. HucMSCs-ex mitigate colitis by suppressing caspase-11/4–mediated pyroptosis in macrophages. Specifically, exosomal miR-203a-3p.2 inhibits caspase-4 activation, while miR-378a-5p dampens NLRP3 inflammasome activity, reducing IL-1β/IL-18 release and limiting pyroptotic cell death (104, 105).

IECs, forming the intestinal epithelium, are essential for the physical and biochemical barrier that segregates host tissues from commensal bacteria to maintain intestinal homeostasis. Disruption of this barrier function, particularly through an imbalance between cellular antioxidant defenses and oxidative stress, is a key driver in the pathogenesis of IBD. As an intracellular antioxidant enzyme specifically localized within mitochondria, Prdx3 confers protection against oxidative stress through the scavenging of mitochondrial ROS. Notably, Loss of the mitochondrial antioxidant enzyme Prdx3 exacerbates DSS-induced colitis through exosomal miR-1260b, which disrupts barrier integrity and enhances proinflammatory signaling (73). In contrast, hucMSCs-ex enriched in miR-129-5p suppress ACSL4-dependent lipid peroxidation, restoring epithelial barrier function and attenuating experimental colitis (106).

Additionally, exosomal miRNAs derived from gut microbiota further shape host immunity. Enterotoxigenic Bacteroides fragilis (ETBF) reduces serum exosomal miR-149-3p via METTL14-mediated m^6^A methylation. The resulting depletion of miR-149-3p promotes Th17 differentiation and accelerates IBD progression, underscoring a direct microbiota–exosome–immune axis (107).

### 4.5 Sjögren’s syndrome

SS is defined by lymphocytic infiltration of salivary and lacrimal glands, leading to progressive glandular dysfunction with xerostomia and keratoconjunctivitis sicca (108). Exosomal ncRNAs critically mediate immune–epithelial crosstalk and aberrant B cell activation in disease pathogenesis.

Activated T cell–derived exosomes in SS transfer miR-142-3p to salivary epithelial cells, suppressing sarco (endo) plasmic reticulum Ca^2+^ ATPase 2b (SERCA2b), ryanodine receptor 2 (RyR2), and adenylate cyclase 9 (AC9). This disrupts Ca²^+^ signaling and cAMP production, impairing protein synthesis and glandular secretion (109). Similarly, EBV-infected B cells deliver exosomal viral miRNA BART13-3p to epithelial cells, where it represses stromal interaction molecule 1 (STIM1) and aquaporin 5 (AQP5). The resulting defects in store-operated Ca²^+^ entry and nuclear factor of activated T cells (NFAT) signaling culminate in reduced saliva secretion (110).

Aberrant B cell activation is another hallmark of SS. Xing et al. investigate the effects of Labial gland mesenchymal stem cells derived exosomes (LGMSCs-ex) on a mouse model of pSS and elucidate their regulatory mechanisms on B cell subsets. Their findings demonstrate that *in vivo* administration of LGMSC-ex to spontaneous pSS mice ameliorates salivary gland inflammatory infiltration and restores saliva secretion. *In vitro*, co-culture of LGMSCs-ex with peripheral blood mononuclear cells (PBMCs) from pSS patients significantly reduces the proportion of CD19^+^CD20^-^CD27^+^CD38^+^ plasma cells. Furthermore, mechanistic studies reveal that miR-125b derived from LGMSCs-ex directly targets PRDM1 in pSS plasma cells. Thus, the miR-125b/PRDM1 axis, by targeting PRDM1 and suppressing plasma cells, represents a promising therapeutic target for pSS (111).

## 5 The application of exosomal ncRNA in autoimmune diseases

### 5.1 Exosomal ncRNAs as diagnostic biomarkers in autoimmune diseases

Exosomal ncRNAs have emerged as promising candidates for early and accurate diagnosis of autoimmune diseases. Their stability in circulation, non-invasive accessibility, and disease-specific expression patterns confer unique advantages over conventional laboratory assays. Across multiple ADs, distinct ncRNA signatures encompassing miRNAs, lncRNAs, and circRNAs have been identified as biomarkers that not only differentiate patients from healthy controls but also correlate with disease activity and therapeutic response. Below, we summarize their diagnostic potential across representative autoimmune disorders.

#### 5.1.1 Rheumatoid arthritis

Conventional serological markers, specifically rheumatoid factor (RF) and anti-cyclic citrullinated peptide (anti-CCP) antibodies, often fail to detect RA in its incipient stages due to limited sensitivity (112). In contrast, exosomal ncRNAs exhibit remarkable stability in circulation, protected from RNase degradation by the lipid bilayer, and display specific expression profiles even during early disease phases. Their levels significantly correlate with disease activity and progression, making them superior candidates for minimizing diagnostic latency and improving accuracy.

As understanding of the critical role of exosomes in RA pathogenesis deepens, exosomal ncRNAs have been identified as promising biomarkers for the early diagnosis and therapeutic management of RA. Wu et al. identify a novel RA-associated microRNA, miR-204-5p, which is enriched in exosomes secreted by human T cells. These exosomes traverses to synovial fibroblasts, suppressing fibroblast proliferation. Furthermore, circulating exosomal miR-204-5p levels are found to be inversely correlated with key clinical parameters, including RF, erythrocyte sedimentation rate (ESR) and C-reactive protein (CRP), suggesting its significant promise as a diagnostic biomarker for RA (113). Similarly, Lu et al. identify reduced plasma exosomal miR-144-3p and miR-30b-5p in RA patients, both negatively correlated with disease activity and anti-CCP levels, with ROC curves showing moderate discriminatory power (AUC = 0.725 and 0.773) (114).

Moving beyond single markers, multi-component panels offer enhanced diagnostic sensitivity. Gong et al. identify a specific three-miRNA signature (miR-885-5p, miR-6894-3p, and miR-1268a) via small RNA sequencing; when combined with anti-citrullinated peptide antibodies (ACPA), this panel achieves a remarkable diagnostic accuracy (AUC = 0.963) (115). Furthermore, a composite panel proposed by Rodriguez-Muguruza et al., comprising exomiR-451a, exomiR-25-3p, and sTWEAK, successfully classifies 95.6% of patients with an AUC of 0.983, underscoring the power of combinatorial approaches for early detection (116).

Beyond miRNAs, dysregulated exosomal lncRNAs and tsRNAs represent emerging diagnostic frontiers. In a study, TCONS_I2_00013502 is significantly elevated, while ENST00000363624 was reduced in RA serum. This elevation corresponds to an area under the curve of 0.870, boasting a sensitivity of 93% and specificity of 68.7%, and 0.864, with a sensitivity of 81.3% and specificity of 78.1%, respectively. Both with strong diagnostic potential (117). Additionally, Li et al. validate two serum exosomal tsRNAs (5’tiRNA-PheGAA and tRF-1-IleAAT) as novel biomarkers, yielding AUC values of 0.836 and 0.876, respectively (118).

#### 5.1.2 Systemic lupus erythematosus

Due to the diversity and substantial immunological heterogeneity of autoantibodies in SLE, the clinical diagnosis of SLE remains challenging. However, the discovery of reliable biomarkers could significantly improve SLE clinical management, thereby improving patient prognosis. Emerging evidence indicates that exosomal ncRNAs offer alternative molecular markers.

The urine exosomal miRNA profile in individuals with SLE exhibits significant differences compared to that of healthy individuals, indicating its potential as a valuable source of renal injury markers (119). Urinary exosomal miR-146a inversely correlates with complement C3/C4, proteinuria, and histological chronicity index, performing well in detecting disease flares (AUC = 0.82). Mechanistically, miR-146a confers protection against inflammation via suppression of IRAK1 and TRAF6 signaling (120). Other urinary miRNAs, including miR-31, miR-107, and miR-135b-5p, differentiate responders from non-responders to therapy, with miR-135b-5p showing the strongest predictive value (AUC = 0.783) (121). Based on a renal miRNA-mRNA co-expression network analysis, Chen et al. also identify and characterize urinary exosomal miR-195-5p as a predictive biomarker for LN (122).

Serum exosomal ncRNAs also exhibit considerable promise as biomarkers for the early detection and prognosis of LN. Studies demonstrate that serum exosomal miRNAs such as miR-497-5p and miR-6515-5p are increased in lupus nephritis, with combined diagnostic performance (AUC = 0.798) (123). MiR-15a-5p is positively correlated with SLE activity and renal involvement, achieving an AUC of 0.81 (124). Meanwhile, lncRNA dysregulation is central to the pathogenesis of SLE. Exosomal lncRNAs LINC00667 and DANCR are significantly upregulated in SLE and correlated with the Systemic Lupus Erythematosus Disease Activity Index 2000 (SLEDAI-2K) scores, with diagnostic AUCs of 0.815 and 0.759, respectively (125).

Novel classes of exosomal tsRNAs also demonstrate strong diagnostic potential. Serum tRF-His-GTG-1 distinguishes SLE from controls with high accuracy (AUC = 0.95 when combined with anti-dsDNA antibodies). Notably, serum tRF-His-GTG-1 alone demonstrates an AUC of 0.81 (sensitivity 66.27%, specificity 96.15%) for differentiating between SLE patients with LN and those without LN, highlighting its value as a non-invasive diagnostic tool (126). Subsequent studies by Chen et al. further explore urinary exosomal tsRNAs as non-invasive biomarkers for LN. Their analysis identifies significantly elevated levels of tRF3-Ile-AAT-1 and tiRNA5-Lys-CTT-1 in urinary exosomes from LN patients compared to both non-LN SLE patients and healthy controls. These two tsRNAs demonstrate distinct diagnostic potential: tRF3-Ile-AAT-1 achieved an AUC of 0.777 (sensitivity 79.63%, specificity 66.69%), while tiRNA5-Lys-CTT-1 yields an AUC of 0.715 (sensitivity 66.96%, specificity 76.92%) (127). Thus, while exosomal ncRNAs in the bloodstream serve as valuable biomarkers for the diagnosis of SLE, their detection in urine offers a more convenient and effective non-invasive method for evaluating disease activity and prognosis.

#### 5.1.3 Type 1 diabetes mellitus

Exosomal ncRNAs are valuable in detecting early β-cell injury in T1DM. EV-associated miR-21-5p is progressively elevated in NOD mice prior to diabetes onset, suggesting its role as an early predictive biomarker (128). In human studies, Pang et al. identify 43 differentially expressed plasma exosomal miRNAs, of which miR-103a-3p, miR-144-5p, and miR-454-3p are validated as diagnostic candidate (129). Additionally, Fan et al. reveal differential expression of plasma-derived exosomal mRNAs in T1DM patients compared with healthy controls. Exosomal mRNA signatures, including ENSG00000198763 (MT-ND2), ENSG00000198786 (MTND5), ENSG00000198840 (MTND3), and ENSG00000269028 (MTTRNR2L12), are also dysregulated in T1DM patients compared to healthy controls and may serve as complementary transcriptional biomarkers (130).

Furthermore, exosomal lncRNAs play key roles in immune regulation. PVT1 and LINC00960, identified within islet exosomal ceRNA networks, modulate inflammatory pathways involving miR-107 and MAPK signaling. The LINC02086-hsa-miR-17-5p-MAPK9/WEE1 and LINC00960-hsa-miR-107-INSIG1 regulatory axes are critically involved in T1DM pathogenesis, representing promising therapeutic targets and diagnostic biomarkers (131). Comprehensive profiling further reveals 162 dysregulated plasma exosomal lncRNAs and 784 circRNAs, with functional analyses implicating circ0005630-miR-1247-5p-ATXN1/ARL6IP1 and circ0007026-miR-324-5p-NCAPD2/PGAM1 networks in T1DM pathogenesis (132, 133). Collectively, these findings highlight exosomal ncRNAs as sensitive biomarkers for both diagnosis and mechanistic insights into T1DM.

#### 5.1.4 Inflammation bowel diseases

Exosomal ncRNAs exhibit differential expression in IBD, suggesting their potential as biomarkers for diagnosis and therapeutic intervention. For example, plasma exosomal miR-149-3p levels are significantly reduced in both UC and CD patients compared to controls, with further decreases observed in active disease relative to remission, indicating its value in monitoring inflammatory activity (107). Moreover, *Fusobacterium nucleatum* infection induces upregulation of exosomal miR-129-2-3p in intestinal epithelial cells, which impairs mucosal barrier function through the TIMELESS-mediated senescence pathway. Elevated serum exosomal miR-129-2-3p is linked with UC, particularly Fn-associated UC, supporting its diagnostic specificity and mechanistic relevance (134).

#### 5.1.5 Sjögren’s syndrome

The current diagnostic criteria for SS rely on characteristic clinical symptoms and relevant autoantibody testing. Due to the insidious onset of SS and the high prevalence of sicca symptoms in the general population, promoting early detection is a paramount objective in contemporary SS research. Exosomal ncRNAs offer new diagnostic avenues. Salivary exosomal miRNAs are first identified as potential biomarkers by Michael et al., while subsequent murine studies reveal upregulation of miR-127-3p, miR-409-3p, miR-410-3p, miR-541-5p, and miR-540-5p in serum exosomes, implicating their role in inflammatory pathways (135, 136).

**Figure 3:**
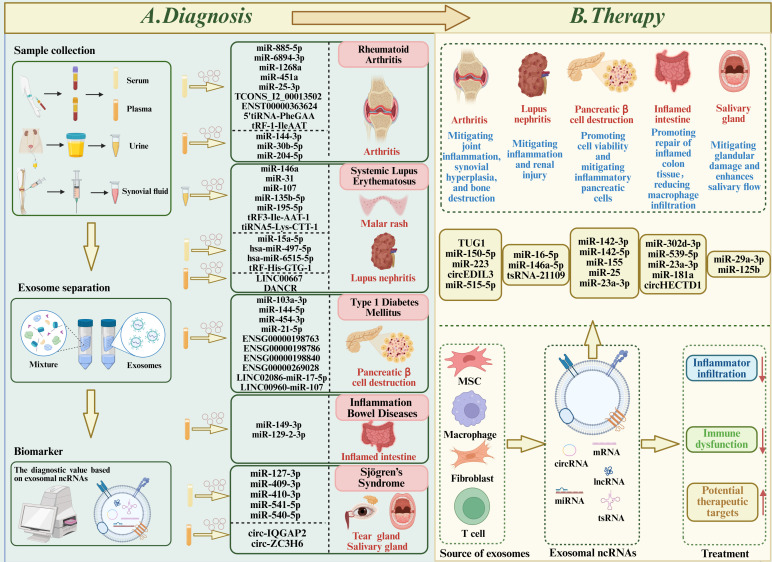
Role of exosomal ncRNAs in autoimmune diseases (ADs). **(A)** Diagnostics: Exosomes from serum, plasma, urine, and synovial fluid of patients with rheumatoid arthritis, systemic lupus erythematosus, type 1 diabetes mellitus, inflammatory bowel disease, and Sjögren’s syndrome show differential expression of ncRNAs. These ncRNAs contribute to disease pathogenesis and are potential biomarkers for early and accurate diagnosis. **(B)** Therapeutics: Exosomes from stem cells, macrophages, fibroblasts, and T cells, administered intravenously or intraperitoneally, and loaded with specific ncRNAs, effectively alleviate disease symptoms and slow progression in experimental models, suggesting translational potential for exosome-based therapies.

More recently, circRNAs gain attention: circ-IQGAP2 and circ-ZC3H6 are significantly upregulated in the minor salivary glands of pSS patients and are validated in plasma exosomes. When integrated with clinical parameters, these circRNAs achieve high diagnostic performance (AUC = 0.92–0.93), underscoring their potential as robust non-invasive biomarkers for SS (137). This section also focuses on other exosomal ncRNAs in SS, which serve as non-invasive diagnostic tools for diagnosing and managing Sjögren’s syndrome effectively (**Table 2****) (****Figure 3**).

**Table 2:** Exosomal ncRNAs as biomarkers of Ads.

Disease	Source	Composition	Biological functions	Ref
RA	Plasma	miR-204-5p	Biomarker	(113)
	Plasma	miR-144-3p, miR-30b-5p	Biomarker	(114)
	Serum	miR-885-5p, miR-6894-3p, miR-1268a,	Biomarker	(115)
	Serum	miR-451a, miR-25-3p, and STWEAK	Biomarker	(116)
	Serum	TCONS_I2_00013502, ENST00000363624	Biomarker	(117)
	Serum	5’tiRNA-PheGAA, tRF-1-IleAAT	Biomarker	(118)
SLE	Urine	miR-146a	Biomarker	(120)
	Urine	miR-31, miR-107, and miR-135b-5p	Biomarker	(121)
	Urine	miR-195-5p	Biomarker	(122)
	Serum	hsa-miR-497-5p, hsa-miR-6515-5p	Biomarker	(123)
	Serum	miR-15a-5p	Biomarker	(124)
	Plasma	LINC00667, DANCR	Biomarker	(125)
	Serum	tRF-His-GTG-1	Biomarker	(126)
	Urine	tRF3-Ile-AAT-1, tiRNA5-Lys-CTT-1	Biomarker	(127)
	Plasma	miR-21-5p	Biomarker	(128)
T1DM	Plasma	miR-103a-3p, miR-144-5p, and miR-454-3p	Biomarker	(129)
	Plasma	ENSG00000198763, ENSG00000198786, ENSG00000198840, ENSG00000269028	Biomarker	(130)
	Plasma	LINC02086-hsa-miR-17-5p, LINC00960-hsa-miR-107	Biomarker	(131)
	Plasma	LncRNA	Biomarker	(132)
	Plasma	CircRNA	Biomarker	(133)
IBD	Plasma	miR-149-3p	Biomarker	(107)
	Plasma	miR-129-2-3p	Biomarker	(134)
SS	Serum	miR-127-3p, miR-409-3p, miR-410-3p, miR-541-5p, miR-540-5p	Biomarker	(136)
	Plasma	circ-IQGAP2 and circ-ZC3H6	Biomarker	(137)

### 5.2 Therapeutic effects and potential of exosomal ncRNAs in autoimmune diseases

Accumulating evidence suggests that exosomal ncRNAs not only act as biomarkers but also play therapeutic roles in autoimmune diseases. By delivering functional ncRNAs, exosomes can modulate immune responses, inhibit inflammatory signaling, restore immune tolerance, and promote tissue repair. This section summarizes current findings on their therapeutic potential in representative ADs.

#### 5.2.1 Rheumatoid arthritis

MSCs-ex demonstrate efficacy in mitigating joint inflammation, synovial hyperplasia, and bone destruction in RA. This therapeutic effect is mediated by the modulation of immune cells. For example, in CIA model mice, hucMSCs-ex enriched with miR-150-5p attenuate bone destruction in CIA mice by suppressing osteoclast differentiation, activating regulatory T cells, and engaging the AhR/CYP1A1 pathway (138). BMSCs-ex deliver lncRNA TUG1, which upregulates BLIMP1 expression, thereby modulating the Th17/Treg cell balance and ultimately ameliorating damage caused by RA (60). MSCs-ex can also modulate the functions of immune cells and synovial fibroblasts, thereby exerting therapeutic effects against RA. BMSCs-exosomal miR-223 inhibits NLRP3 inflammasome activation and reduced IL-1β, TNF-α, and IL-18 production, thus ameliorating joint inflammation (139). Exosomal circRNAs play critical roles in RA. Exosomes derived from synovial mesenchymal stem cells (SMSCs-ex) derived circEDIL3 suppress pathological angiogenesis through the miR-485-3p/PIAS3/STAT3/VEGF axis, reducing arthritis progression (140).

Furthermore, mitochondrial dysfunction is increasingly recognized as a pivotal driver of RA pathogenesis. Notably, the crosstalk between compromised mitochondrial integrity and pyroptosis establishes a vicious cycle that perpetuates synovial inflammation and joint damage (141). Consequently, restoring mitochondrial homeostasis represents a vital strategy for preserving cellular viability and curbing inflammation. In this context, BMSCs-ex ameliorate RA pathology by delivering miR-515-5p. Mechanistically, miR-515-5p directly targets TLR4 to inhibit NLRP3 inflammasome activation and subsequent pyroptosis, thereby preserving mitochondrial function and attenuating joint destruction (142). Taken together, these findings underscore the capacity of exosomal ncRNAs to act as potent therapeutic mediators in RA, highlighting their significant potential as novel targets for clinical intervention.

#### 5.2.2 Systemic lupus erythematosus

The related signaling pathways and proteins modulated by exosomal ncRNAs in SLE holds significant potential for uncovering novel pathological mechanisms, thereby facilitating the development of improved clinical diagnostic and therapeutic strategies. Exosomes serve as effective delivery vehicles for ncRNAs, enabling targeted downregulation of gene expression and thereby potentially treating diseases. Zhang et al. find that ADSC-ex delivering miR-16-5p downregulated LATS1, restoring immune homeostasis (59). HucMSCs-ex carrying miR-146a-5p suppress NOTCH1 expression, induce M2 macrophage polarization, and alleviate diffuse alveolar hemorrhage (66). Furthermore, Additionally, MSC-exosomal tsRNA-21109 inhibits M1 polarization and pro-inflammatory cytokine release, thereby mitigating disease severity (143). Collectively, these findings suggest that exosomal ncRNAs can modulate innate immune responses and offer therapeutic potential for SLE and lupus nephritis.

#### 5.2.3 Type 1 diabetes mellitus

Exosomal ncRNAs mediate communication between β-cells and immune cells, influencing T1DM progression. On one hand, exosomes released from T lymphocytes (containing miR-142-3p, miR-142-5p, and miR-155) transfer to pancreatic β-cells and induce apoptosis, representing a novel mechanism driving T1DM pathogenesis (85).

On the other hand, T1DM pathogenesis is characterized by an imbalance between autoreactive T cells and Tregs (144). Qu and colleagues demonstrate that gingival mesenchymal stem cells (GMSCs)-ex treatment rectifies this immune imbalance by suppressing Th1/Th17 expansion and promoting Treg subsets, including CD4^+^CD25^+^Foxp3^+^ Tregs and IL-10-producing type 1 regulatory T (Tr1) cells. Mechanistically, GMSCs-ex delivers miR-23a-3p targeting IL-6R, thereby restoring immune balance and alleviating insulitis in T1DM (145). Moreover, Exosomal miR-25 from MSCs can attenuate the infiltration of activated T lymphocytes into the pancreas in T1DM by suppressing CXCR3 expression, thereby alleviating disease progression (102). These results highlight exosomal ncRNAs as key regulators of immune–β-cell interactions and promising therapeutic tools in T1DM.

#### 5.2.4 Inflammation bowel diseases

MSCs-ex holds significant therapeutic potential due to their ability to mimic the biological functions of MSCs in promoting tissue repair, suppressing local inflammation, and modulating the immune system. Research demonstrated that HucMSCs-ex enriched with miR-302d-3p inhibits FLT4/VEGFR3/AKT signaling, thereby reducing lymphangiogenesis and macrophage infiltration in colitis models (146). BMSCs-ex carrying miR-539-5p suppresses NLRP3-mediated pyroptosis and ROS accumulation in intestinal epithelial cells (147).

Beyond immunomodulation, exosomal ncRNAs are pivotal in restoring barrier integrity. For instance, miR-23a-3p-enriched exosomes from human amniotic epithelial stem cells (hAESCs) promote mucosal repair by downregulating TNFR1 and suppressing NF-κB activation (148). Li et al. further reveals that MSCs-derived exosomal miR-181a exerts protective effects in DSS-induced colitis, partially by modulating the gut microbiota and enhancing barrier function (149). Epigenetic regulation, particularly m^6^A methylation, also offers a novel avenue. BMSCs-derived exosomal circHECTD1 binds CTCF to inhibit METTL3 transcription. This suppression reduces m^6^A methylation of *claudin1* mRNA, thereby enhancing Claudin-1 expression and reinforcing the intestinal barrier to alleviate UC (150). Together, these findings indicate that exosomal ncRNAs play both protective and pathogenic roles in IBD, representing dual therapeutic targets.

#### 5.2.5 Sjögren’s syndrome

MSCs-ex deliver diverse molecular cargo, including regulatory ncRNAs that modulate immune pathways relevant to SS therapy. For example, exosomes derived from stem cells from human exfoliated deciduous teeth (SHED-ex) enriched with miR-29a-3p suppress Th1 differentiation via the T-bet pathway, improving salivary secretion (151). LGMSCs-ex delivering miR-125b attenuate experimental SS by targeting PRDM1, which suppresses plasma cell differentiation and reduces glandular injury, suggesting their therapeutic potential in pSS (111) (**Table 3****) (****Figure 3**).

**Table 3:** Therapeutic applications of exosomal ncRNAs in Ads.

Disease	Source	Composition	Function	Ref
RA	BMSC	lncRNA TUG1	Modulate the Th17/Treg cell balance and ameliorate damage	(60)
	HucMSC	miR-150-5p	Activation of the AhR/CYP1A1 signaling pathway	(138)
	BMSC	miR-223	Suppress NLRP3 activation	(139)
	SMSC	circEDIL3	Suppress inflammation-induced angiogenesis via miR-485-3p/PIAS3/STAT3/VEGF axis	(140)
	BMSC	miR-515-5p	Improve pyroptosis and mitochondrial integrity through TLR4/NLRP3/GSDMD axis	(142)
SLE	ADSC	miR-16-5p	Modulate immune responses by the miR-16-5p/LATS1 axis	(59)
	HucMSC	miR-146a-5p	Promote M2 macrophage polarization	(66)
	HucMSC	tsRNA-21109	Inhibit M1 macrophage polarization	(143)
T1DM	Lymphocyte	miR-142-3p, miR-142-5p, miR-155	Promote pancreatic β cell death	(85)
	MSC	miR-25	Inhibit T cells migration	(102)
	GMSC	miR-23a-3p	Target IL-6R and suppress Th1/Th17 expansion and promoting Treg subsets	(145)
IBD	HucMSC	miR-302d-3p	Regulate lymphangiogenesis via the miR-302d-3p/VEGFR3/AKT axis	(146)
	BMSC	miR-539-5p	Inhibit pyroptosis through NLRP3/caspase-1 signaling	(147)
	hAESCs	miR-23a-3p	Promote colonic recovery via MiR-23a-TNFR1-NF-κB Signaling pathway	(148)
	MSC	miR-181a	Modulate the gut microbiota and enhancing intestinal barrier function	(149)
	BMSC	circHECTD1	Regulate the balance of gut microbiota and Th17/Treg cells and alleviate UC	(150)
SS	LGMSC	miR-125b	Target PRDM1 and Suppress Plasma Cells	(111)
	SHED	miR-29a-3p	Suppress Th1 cell differentiation	(151)

## 6 Summary and prospect

Over the past decades, intensive studies have revealed the pivotal roles of exosomal ncRNAs as regulatory molecules and promising biomarkers in autoimmune diseases, underscoring their translational potential for both clinical diagnosis and therapeutic intervention. This review delineates the structural features of exosomes, the heterogeneity of their non-coding RNA cargoes, and their multifaceted regulatory mechanisms. Specifically, exosomal ncRNAs coordinate pathogenic processes in autoimmune diseases by modulating immune responses, oxidative stress, autophagy and cell cycle dysregulation (9). Furthermore, our analysis establishes the critical interconnectedness of these pathways, highlighting a positive feedback loop between exosomal ncRNA-mediated immune activation and ROS accumulation. Additionally, this review encompasses recent advances in tsRNAs as diagnostic markers (e.g., serum tRF-His-GTG-1 in SLE) and analyzes the role of m^6^A modification in regulating exosomal ncRNAs in IBD. Exosomal ncRNAs exhibit differential expression across various diseases, suggesting their potential as biomarkers for early detection.

Moreover, this review integrates diagnostic biomarkers and therapeutic strategies, offering a complete translational research framework. As biomarkers, exosomal ncRNAs display distinct advantages over their non-exosomal counterparts. The lipid bilayer of exosomes protects encapsulated ncRNAs from RNase-mediated degradation in biological fluids, thus enhancing their stability and preserving their integrity (152). In addition, exosomes actively enrich ncRNAs, resulting in higher concentrations in biological fluids (e.g., blood, urine, saliva) compared with freely circulating ncRNAs. This enrichment facilitates more sensitive and accurate detection (153). Furthermore, since exosomal ncRNAs originate from specific cell types, they faithfully reflect the molecular signature of their parental cells, thereby providing enhanced tissue specificity and pathological relevance (154). Aberrant expression profiles across different ADs highlight their potential utility in early diagnosis, disease classification, and therapeutic monitoring. From a therapeutic perspective, exosomal ncRNAs offer multiple advantages. Functionally, they can regulate inflammation-related pathways and modulate immune homeostasis, making them versatile therapeutic agents (155). Structurally, exosomal encapsulation protects ncRNAs from rapid enzymatic degradation, ensuring stability and efficient systemic delivery (152). Moreover, exosomes can be engineered as delivery vehicles to encapsulate therapeutic ncRNAs, such as siRNAs, and targeted to specific tissues, further enhancing treatment precision and efficacy (156).

Although exosomal ncRNAs demonstrate considerable potential as diagnosis biomarkers and therapeutics for diverse ADs, key translational barriers hinder their clinical adoption. Primarily, the therapeutic adequacy of purified exosomes remains to be rigorously evaluated, particularly regarding their ability to recapitulate beneficial effects observed in preclinical models. Secondly, while most studies focus on single ncRNA species, the coordinated impact of exosomal miRNA-lncRNA-circRNA networks remains largely unexplored. CircRNAs, given their stability and sponge functions, may act as core regulators within these networks, warranting further mechanistic validation. Thirdly, the translational potential of exosomal ncRNAs is hindered by technical challenges in exosome isolation standardization, ncRNA cargo characterization, and targeted delivery. Current studies rely heavily on animal models, and clinical evidence from large-scale cohort studies is lacking, particularly regarding the diagnostic and prognostic value of exosomal ncRNAs as biomarkers. Finally, the interplay between exosomal ncRNAs and environmental factors in autoimmune initiation remains a promising yet under-investigated direction.

Notably, a critical area for future research lies in elucidating the mechanisms underlying the marked gender bias in autoimmune diseases, which predominantly affect females. Evidence indicates that distinct gender-specific signatures of the XIST lncRNA are present in systemic SLE. Specifically, XIST, expressed exclusively in females to randomly inactivate one of the two X chromosomes for gene dosage compensation, acts as an endogenous female-specific danger signal. During cell death, XIST-protein complexes (RNPs) are released within EVs. Subsequently, the packaged XIST RNA stimulates Toll-like receptor 7 (TLR7)-dependent interferon (IFN) secretion by plasmacytoid dendritic cells (pDCs), thereby promoting SLE development and exacerbating disease activity (157). This finding has broad implications for understanding the molecular basis of female susceptibility to autoimmunity. However, studies systematically profiling gender-specific exosomal ncRNAs in other autoimmune diseases remain scarce. Future research with larger cohorts and a detailed focus on which XIST-related antigens contribute to female-biased immunity will be valuable.

In conclusion, exosomal ncRNAs represent a rapidly emerging frontier in autoimmune disease research, with great promise for improving diagnostic precision and therapeutic innovation. Overcoming current translational challenges through systematic mechanistic studies, rigorous validation in large populations, and development of standardized detection and engineering strategies will be critical to unlocking their full clinical potential. Ultimately, exosomal ncRNAs may provide a foundation for personalized medicine approaches in the management of autoimmune diseases.
